# An integrative measure of cognitive performance, but not individual task performance, is linked to male reproductive output in budgerigars

**DOI:** 10.1038/s41598-021-91213-3

**Published:** 2021-06-03

**Authors:** Angela Medina-García, Timothy F. Wright

**Affiliations:** 1 Department of Ecology and Evolutionary Biology, University of Colorado at Boulder, 334 UCB, 1900 Pleasant Street, Boulder, CO 80309 USA; 2 Department of Biology, New Mexico State University, MSC 3AF, 1200 Horseshoe Drive, Las Cruces, NM 88003 USA

**Keywords:** Sexual selection, Animal behaviour

## Abstract

Cognitive abilities such as learning and memory are key for survival and reproduction. Individuals with high cognitive abilities may be more successful at attracting mates and producing offspring. However, empirical tests of and evidence supporting this hypothesis remain scarce. We measured cognitive performance of male budgerigars in four tasks: problem solving, detour reaching, seed discrimination, and spatial memory. We then tested female choice for male cognition at three stages of the mating choice process: social pairing, extra-pair mating, and continued reproductive investment with a social mate. We also measured female reproductive output. We used an integrative measure of male cognitive performance that encapsulates performance across all tasks, the ‘composite cognitive score’ by summing performance on the four tasks. In the first stage, females did not choose their social mates based on any of the measures of male cognitive performance. In the second stage, however, males with higher composite cognitive scores sired and raised more offspring. In the third stage, females increased their reproductive investment after the first breeding attempt when paired with males with higher detour-reaching scores. These results suggest that female reproductive decisions may shape overall male cognitive performance.

## Introduction

Cognitive processes underlie the successful execution of many behaviors critical for an animal’s survival and reproduction, including foraging, predator avoidance, and parental care^1^. Individual variation in cognitive abilities has been extensively documented across taxa^2^. Furthermore, evidence supporting the heritability of cognitive traits in non-human vertebrates and invertebrates is mounting^3^. Cognitive traits are likely to be under selection if these individual differences, besides being heritable, also result in differential fitness^4,5^. The nature and strength of selection on cognitive abilities, however, remains an outstanding question for behavioral and cognitive ecologists.

It was Darwin who first suggested that “intelligence” or cognitive traits could be subject to sexual selection^6^. Sexual selection, defined as differential reproductive success caused by either intrasexual selection (male–male competition) or intersexual selection (female choice)^7^, represents a plausible mechanism for the evolution of cognition^8^; but it remains unclear as to how ubiquitous this selection is and how it shapes cognitive traits. Some have suggested that what males are truly displaying during courtship displays are their cognitive skills^9^. Male song in oscine birds is one example of how a sexually selected display could be a reliable indicator of brain development and cognitive abilities^9,10^. If females can reliably evaluate cognitive abilities in males through these displays, and if these abilities are heritable, then females can obtain indirect fitness benefits by mating with males with high cognitive abilities by either producing attractive offspring^11^ or by producing offspring with “good genes”^12^. Furthermore, when males contribute to the raising of offspring, as in socially monogamous species, females also can obtain direct benefits through male parental care. It is reasonable to assume that superior cognitive abilities in males can directly improve offspring survival and quality through an enhanced ability to obtain resources for provisioning of the offspring and coordination of reproductive efforts^8^. Direct evidence of female mate choice for male cognition in non-human vertebrates, though, is limited and mixed^8,13,14^, particularly in monogamous species.

Parrots (Order Psittaciformes) show remarkable cognitive abilities from problem solving to vocal matching^15,16^. Budgerigars (*Melopsittacus undulatus*) are gregarious parrots that form large nomadic flocks in search for ephemeral food and water sources across Central Australia^17^. In captivity, they are socially monogamous and form long-term pair bonds^18^. Females are socially dominant and aggressive, choosing which males they allow to court them^18^. Males contribute heavily to raising the offspring by either feeding the females when the chicks are young, or feeding the nestlings directly once they are older^19^. A recent study has suggested that female budgerigars are attracted to males with superior problem-solving abilities^20^. However, it is still unknown whether such preferences would actually translate into actual mate choice or fitness benefits.

We investigated female mate choice for male cognitive abilities within a novel multistage mate choice process. In socially monogamous species with biparental care, females initially choose a social mate to form a pair bond (first stage of choice). During breeding, females choose to copulate with certain males outside of the pair bond (second stage of choice). As the female gains more information about the parental performance of her social mate, she may invest more or less in reproduction by either modulating the number of eggs laid or by deciding whether or not to produce more broods after the first brood (third stage of choice). We assessed male cognitive abilities in terms of performance in a foraging problem, a spatial memory task, a seed discrimination task, and detour-reaching task. We also calculated an overall measure of performance that integrated performance in all the tasks, the composite cognitive score. We then tested female choice at these three stages, and female reproductive output. We found that female budgerigars obtain direct fitness benefits from mating with males with higher composite cognitive scores. Our results provide support for the longstanding hypothesis that sexual selection is a viable mechanism for the evolution of cognition.

## Results

### Cognitive performances

**Figure 1:**
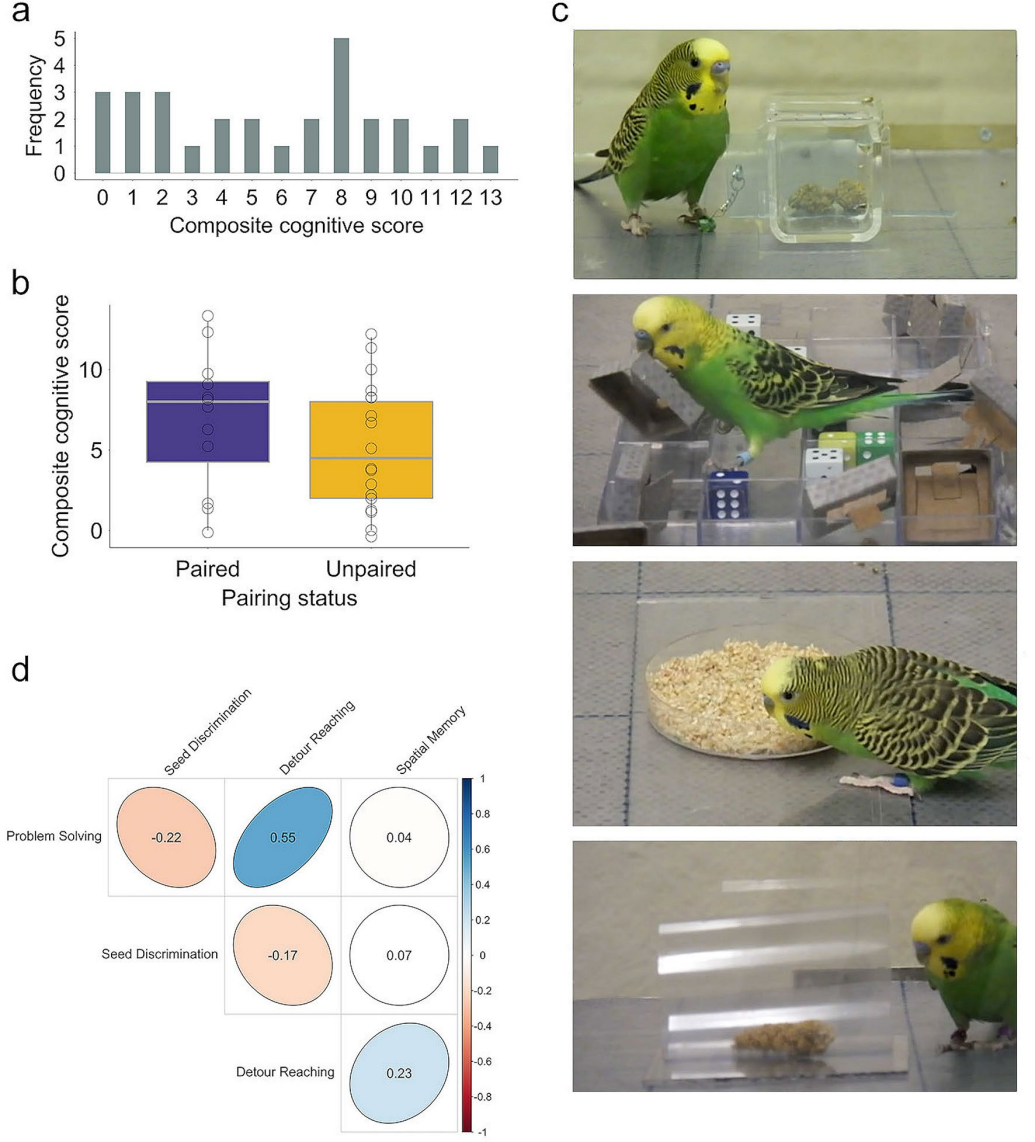
Females do not pair with social mates based on cognitive performance. (**a**) Distribution of the composite cognitive score of budgerigar males (*N* = 30). (**b**) Composite cognitive scores did not differ between unpaired (*N* = 18) and paired males (*N* = 12) at the stage of choice for a social mate. Open circles represent each male, bars indicate the median across individuals in each group. (**c**) Devices employed to assess cognitive abilities in male budgerigars. Top to bottom: problem-solving task; setup for testing spatial memory (colored plastic served as landmarks); seed discrimination task (seed husks were glued to the petri dish); detour-reaching task. (**d**) Spearman rank correlations between performance in the 4 cognitive tasks. The cognitive measures compared were problem-solving score, maximum seed discrimination efficiency, detour-reaching score, and average number of errors in the spatial memory task. Blue indicates positive associations and red indicates negative associations. Numbers inside bubbles are the correlation coefficients. Number of pairwise comparisons ranged between 12 and 27. Correlations were not significant after the Bonferroni correction (α level of significance = 0.05/6 = 0.008).

We tested and calculated the performance of 30 adult male budgerigar males in four cognitive tasks: problem solving, spatial memory, seed discrimination, and detour reaching. Performances among tasks were not collinear (Fig. 1d; r < 0.7)^21^, consistent with previous results in a larger set of budgerigar males^22^. These weak and non-significant correlations among performance in different tasks suggest that each of the tasks that we employed reflects abilities in a different cognitive domain. In such cases a measure of cognitive performance that weights performance across all tasks equivalently is a better measure of integrative performance than a single factor from a Principal Component Analysis on task performances. We calculated this integrative measure of performance as a composite cognitive score, similar to an overall score in a standardized test (for details on the calculation of this score, see the “Materials and methods” section). Males exhibited a wide individual variation in performance in each task (Table S1 in the “Supplementary Information”) as well as in composite cognitive score (Fig. 1a).

### First stage: females do not choose social mates based on cognitive performance

In order to test female choice for a social mate, and therefore male pairing success, we used a replicated free-pairing experiment that consisted of 5 mixed-sex groups of a 2:1 male-biased ratio (n = 6 males and 3 females per group) (see “Materials and methods” for details), compared to the sex ratio of 1:1 found in natural populations^23^. Considerable variation in cognitive performance was found within each of the free-pairing groups (Table S2 in the “Supplementary Information”). At this stage, 12 males successfully formed stable pair bonds with females and 18 males remained unpaired. Stable pairs formed between 9 and 76 days after the pairing experiment started (average ± SE: 42.83 ± 6.49 days).

None of the measures of task performance (including the composite cognitive score), nor the covariates measured (personality score, body condition index), had a significant effect on male pairing success (Fig. 1b, Table S3 in the “Supplementary Information”). This was the case for the data set with all males (generalized linear model (GLM): *z* = − 0.292, *b* = 0.65, *P* = 0.77) (Fig. 1d), and the data set where six males who lacked data on aggressiveness and sociability (generalized linear model (GLM): *z* = − 0.633, *b* = 1.066, *P* = 0.527) (*N* = 24). The first stable pair in the group of these six males formed within the first week of the pairing experiment, so we were unable to collect one full week of data corresponding to their interactions prior to pair formation (see details on construction of social networks in “Supplementary Information”). Among paired males, the composite cognitive score was not a predictor of the time to form a stable pair (*R*^2^ = 0.03, *R*^2^_adj_ = − 0.07 *F*_1,10_ = 0.04, *P* = 0.60). We found no support for assortative pairing based on plumage (Sign test: *number of pairs with matching plumage* = 9, *number of pairs* = 12, *P* = 0.146).

### Second stage: males with higher cognitive scores sired more offspring

We allowed 45 adults (30 males and 15 females) that participated in the free-pairing experiment to breed in an aviary for 6 months to examine the second stage of pairing. All birds were genotyped using microsatellite markers, and paternity of offspring was assigned by calculation of the likelihood ratio scores (LODs) (see “Materials and methods” and “Supplementary materials and methods” for details). We were able to assign, with confidence, genetic paternity to 72 of the 83 resulting nestlings (87%). Social fathers were the genetic fathers of 33.3% of these nestlings, while 66.7% were fathered by extrapair males. Twenty-five genetic fathers were identified for the 48 extrapair offspring. Fourteen of these (56%) were paired to other females. Overall, 60% of males sired at least one within-pair nestling in their own nests.

**Figure 2:**
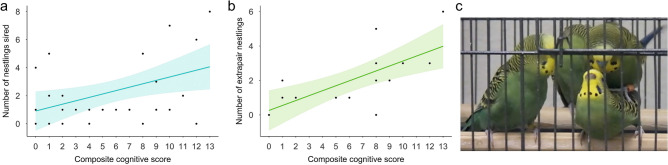
Males with higher composite cognitive scores sire more nestlings. (**a**) Relationship between male composite cognitive score and number of nestlings sired (*N* = 30). (**b**) Relationship between male composite cognitive score and number of extra-pair nestlings. Only males that were paired during the breeding phase were included (*N* = 15). Shaded areas represent 95% confidence intervals from the generalized linear models. (**c**) A female budgerigar engaged in an extra-pair copulation next to her social mate (left).

We found a significant effect of male composite cognitive score on both the total number of nestlings sired by males (Fig. 2a Table 1), and on the number of extra-pair nestlings produced by paired males (Fig. 2b,c, and Table 1). Among individual cognitive task performances, only problem-solving score had a significant effect on the number of nestlings sired (Table S4 in the “Supplementary Information”). None of the individual task performances or any of the covariates had an effect on the number of extra-pair nestlings (Table S5 in the “Supplementary Information”).

**Table 1:** Effects of male composite cognitive score, male plumage morph, and male body condition index (BCI) on number of nestlings sired and number of extrapair nestlings.

Response variable	Data Set	Explanatory variables	Estimate (mean ± SE)	*Z* value	*p*-value
Number of nestlings sired	All males (*N* = 30)	Intercept	0.559 ± 0.912	0.613	0.540
		**Composite cognitive score**	**0.099 ± 0.032**	**3.102**	**0.002**
		Male plumage morph (OP)	0.382 ± 0.422	0.905	0.365
		Male plumage morph (WT)	0.347 ± 0.371	0.936	0.349
		BCI	− 0.005 ± 0.006	− 0.820	0.412
Number of extrapair nestlings	Paired males (*N* = 15)	Intercept	− 1.065 ± 1.146	− 0.929	0.353
		**Composite cognitive score**	**0.141 ± 0.051**	**2.761**	**0.006**
		Male plumage morph (OP)	0.384 ± 0.679	− 0.567	0.571
		Male plumage morph (WT)	1.007 ± 0.563	1.787	0.074
		BCI	4.742 × 10^–3^ ± 0.006	0.074	0.941

Significant results are indicated in bold.

### Third stage: male composite cognitive score does not affect female reproductive investment

We estimated female reproductive investment with her social mate as the number of eggs that a female laid on the first nesting attempt and subsequent attempts, both separately and combined. We also estimated female investment as the number of nesting attempts throughout the breeding period. The detour-reaching score of individual males influenced the investment of the female in terms of eggs laid after the first nesting attempt (Fig. S1 and Table S6 in the “Supplementary Information”), but there was no effect of composite cognitive score, nor performance on the other three tasks (Fig. S1 and Table S6 in the “Supplementary Information”). Interestingly, females paired with males that performed poorly in the detour-reaching task, laid significantly more eggs in their first nesting attempt (Table S6 in the “Supplementary Information”). However, after the first nesting attempt, the trend was the opposite: females paired with males that performed well in the detour-reaching task laid more eggs than did those paired with males with low detour-reaching scores (Fig. S1 in the “Supplementary Information”). Male cognitive performance measured with the composite cognitive score or performance in specific cognitive tasks did not impact significantly the number of nesting attempts by the pair, or the total number of eggs that a female laid over the course of the breeding season (Table S7 in the “Supplementary Information”).

### Fitness consequences: females paired with males with high cognitive performance fledge more nestlings

**Figure 3:**
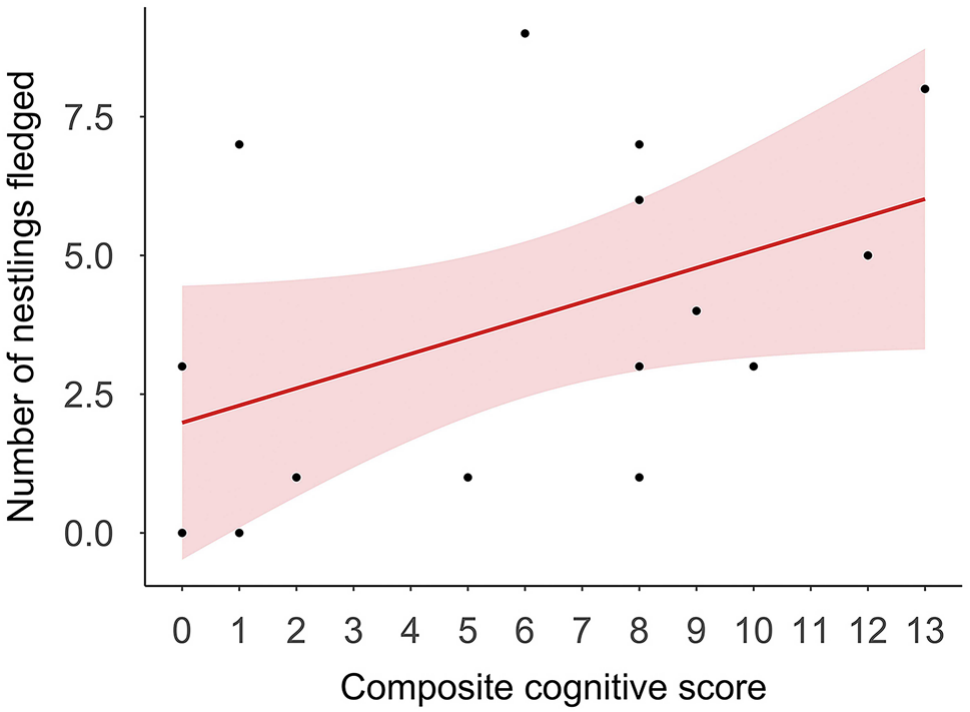
Males with higher composite cognitive score raise more nestlings. Relationship between male composite cognitive score and number of nestlings fledged (*N* = 15). Shaded areas represent 95% confidence intervals from the generalized linear models.

During the six months that the 45 adults bred, established pairs attempted to nest between 1 and 6 times. During this period, 15 males attempted to raise nestlings with their social mates, and 4 of these males shared the parental care with another male at the same nest (*N* = 2 nests). In one instance, two pairs used the same nest box, and both contributed to raise the nestlings from that nest. In the other instance, a male that was initially paired with another female, abandoned that female and paired with an already paired female, raising offspring jointly with her other mate. In the case that two males shared the parental care, half of the nestlings fledged at the nest were assigned to each male. Our analyses revealed that male composite cognitive score had a significant effect on the number of nestlings fledged (Fig. 3 and Table S8 in the “Supplementary Information”). These results held after removing the four males with shared parental care (Table S8 in the “Supplementary Information”). Performance in the spatial memory task had a significant effect on the number of nestlings fledged (Table S9 in the “Supplementary Information”), however this relationship was no longer significant when the four males with shared parental care were excluded from the model (generalized linear model (GLM): *z* = − 1.407, *b* = 1.952, *P* = 0.160).

## Discussion

Our study provides evidence for the role of sexual selection in the evolution of cognition. We tested female mate choice in budgerigars for male cognitive abilities using a novel multistage mate choice process that considers the complexity of such choice in socially monogamous species with biparental care. We found evidence of female mate choice for male cognition at the level of extra-pair paternity and female reproductive investment. Female budgerigars also increased their reproductive investment after the first breeding attempt when paired with males that performed well in the detour-reaching task. Furthermore, males with higher composite cognitive scores raised more nestlings, which may translate into direct and indirect fitness benefits for their social mates. While these results are derived from a captive population of limited size, we believe they offer some important general insights into the evolution of cognitive abilities.

Our results show that males with higher general cognitive performance sire more offspring, which illustrate the evolutionary benefits of female male choice for male cognition. These results are consistent with those previously found in the polygamous satin bowerbirds^13^. In the case of budgerigars, it seems quite plausible that females assess the attractiveness of males with higher cognitive abilities through direct observation of male performance, as recently demonstrated in a separate study that assessed budgerigar female preferences^20^. During the breeding stage of our study, while the birds reproduced in the aviary, females had ample opportunities to observe and assess cognitive abilities that allowed males to effectively obtain food resources for both mates and nestlings. While it lacks some of the challenges of life in the wild, the aviary setting does represent a socially complex environment, with some level of spatial and temporal variation of food availability (see “Supplementary Information”). In this setting, females could assess a male’s cognitive processes underlying competitive ability such as individual recognition, learning, and memory, as well as processes related to detection and extraction of high-quality food resources. It is also possible that males with better cognitive abilities are better at obtaining extra-pair copulations while evading detection and possible retribution by their mates^24^. As discussed below, these opportunities might not be present during the earlier mate choice stage of the mating process, where we did not see an effect of male cognitive ability on female choice.

At the third stage of female mate choice, continued reproductive investment, we found evidence for increased reproductive effort after the first nesting attempt based on male performance in the detour-reaching task. Interestingly, females paired with males with higher detour-reaching scores, laid fewer eggs in the first nesting attempt. One plausible explanation for this result is that, during the first nesting attempt, female reproductive investment is determined by other indicators of male cognitive ability, such as vocal performance, that were not measured in this study but might be inversely correlated to detour-reaching performance. There is evidence suggesting tradeoffs between cognitive abilities in song sparrows, *Melospizia melodia*, in which there is an inverse relationship between repertoire size and speed learning in a spatial memory task^25^. In subsequent nesting attempts, however, female budgerigars in our study that paired with males with higher detour-reaching scores laid more eggs. We propose that after one nesting attempt, females have already thoroughly assessed their social mate’s performance via nestling provisioning, for instance, which directly reflects male’s ability to locate and efficiently exploit food sources based on his cognitive abilities. Females may control the number of eggs produced by simply controlling the frequency of copulations and therefore fertilizations, or by a number of postcopulatory mechanisms, including sperm rejection^26,27^.

Our results suggest that females potentially obtain indirect fitness benefits from mating with males with higher cognitive performance, by producing male offspring with high cognitive abilities that would in turn be more attractive mates. A study in the western subspecies of Australian magpies, *Cracticus tibicen dorsalis*, showed that female general cognitive performance has a positive impact on reproductive success^28^. However, the significant effect of male cognitive score on number of nestlings fledged that we found supports the idea that females also obtain direct fitness benefits (i.e. more offspring) from pairing with males that show high overall cognitive performance. Similar results were found in wild house sparrows, *Passer domesticus*, where problem-solving males fledged more nestlings than non-solvers^29^ and in New Zealand robins, *Petroica longipes*, where males with a more accurate memory produced more fledglings^30^. However, our study further strengthens the link between male cognition and reproductive success in several important ways. First, we employed an integrative measure of male cognitive performance, the composite cognitive score, which reflected performance across multiple cognitive tasks that likely draw on different cognitive domains. One of the issues with employing success or failure in a single cognitive task is the potential confounding effect of non-cognitive factors such as personality, as shown previously in a larger set of male budgerigars, which included the test subjects in this study^22^. It is likely that a male’s ability to raise offspring is impacted by a combination of cognitive abilities in different domains, which explains why our composite score is related to male fitness while individual task performances are not. Second, we were able to control for the stochastic effects of predation on reproductive success, which allowed us to isolate the impact of male cognitive performance on reproductive success.

The results from our comprehensive multistage mate choice process approach revealed female choice for male cognitive abilities at advanced stages of the pair bonding, rather than at the initial choice for a social mate. These results could be explained by the biology and ecology of our study species, and are consistent with the results in a study in budgerigars that evaluated the role of vocal learning during female choice for a social mate as well as choice for extra-pair mates^31^. Budgerigars reproduce opportunistically when food supply is abundant^32^. In consequence, females may have to make quick decisions when selecting a mate, based for example, on the syntactical consistency of male warble song^33^, or other rapidly-assessed indicators of male quality such as male UV plumage reflectance^34,35^. In this scenario, females may be unable to assess valuable cognitive skills in a mate such as the ability to remember food sources (i.e. spatial memory), discriminate efficiently among food sources of different nutritional value (i.e. seed discrimination), being able to solve foraging problems (i.e. problem-solving abilities and detour-reaching performance), or the ability of a male to learn its mate’s contact call^16,31^. In species in which the onset of reproduction is not as sudden as in budgerigars, such as migratory species that arrive to the breeding grounds weeks before they start breeding, or species in which males are territorial year-round, and cognitive abilities might determine resource holding potential, females may have more opportunities to evaluate male cognitive skills prior to selecting a social partner.

Another explanation for our results at the first stage of choice is that pairing by males and females may be assortative in regard to cognitive abilities. Female mate choice itself involves cognitive processes, from discrimination through decision making. Therefore, female mate choice is not only dependent on male performance, but also on female’s ability to evaluate high-quality males^20,36,37^. For instance, experimental evidence from zebra finches, *Taeniopygia guttata*, has shown that low-quality females prefer low-quality males and only high-quality females showed a preference for high-quality males^38^. Where it occurs, such assortative mating could blunt any directional selection for male cognitive abilities. We recommend that future tests of mate choice for male cognition also evaluate the role of female cognitive abilities in this choice. Although female budgerigars are highly aggressive and selective as to which males they permit to court them, it is also possible that males are selective as to which females they attempt to court. This could also explain why we did not see any relationship between male cognitive performance and female choice at the initial mate choice stage of choice for a social mate.

Humans are the only species to date for which there is consistent evidence supporting female mate choice for male cognition^39^. Budgerigars are similar to humans in having a disproportionately large brain relative to their body size and being socially monogamous with biparental care. These commonalities between humans and budgerigars, along with our findings, lead us to propose that both monogamous mating systems and male parental care are important conditions driving the evolution of cognition via sexual selection. Our results suggest that females obtain direct fitness benefits from raising offspring with males with higher cognitive abilities and may also obtain indirect fitness benefits from mating with these males. Furthermore, females seem to adjust their investment in reproduction according to certain cognitive abilities exhibited by their mates. Together, these results provide support for sexual selection as a plausible mechanism for the evolution of cognition under some conditions.

## Materials and methods

### Subjects and housing conditions

The study subjects were 30 domesticated male budgerigars and 15 females purchased from a wholesale breeder (McDonald Bird Farm) at the age of 2–3 months. Per the breeder, study subjects experienced generally similar environmental conditions during rearing, including ad libitum access to food and water. Each individual was banded metal band with a unique number and a color plastic band. Males and females were purchased separately and housed in separate rooms until the mate choice phase of the experiment. Birds were housed in groups of 10–12 individuals in flight cages (79 × 52 cm, and 135 cm high) under a 12:12 light:dark cycle, under standard fluorescent lighting, at a room temperature of 24 ± 2 °C. Birds had multiple wooden perches of different thickness and commercial captive bird toys in their cages, as well as parakeet seed mix, cuttlebone, and vitamin water ad libitum.

All individuals had identical history of experience with the materials that composed the cognitive devices prior to testing. For further details on experimental animals, housing conditions, and general experimental setup, see the “Supplementary Information”.

The reporting in this manuscript follows the recommendations in the ARRIVE guidelines.

### Ethics approval

Budgerigar care was in accordance with guidelines by the Animal Care Committee of the New Mexico State University. All experimental protocols were approved by the Animal Care Committee of the New Mexico State University (IACUC protocol 2013-030). All the experiments in this study were performed in accordance with guidelines and regulations by the institutional IACUC committee.

### Problem-solving task

The goal of this task was to assess individual ability to learn how to solve a foraging problem, in this case, moving a clear plastic barrier to obtain a reward. We believe that performance in this task may reflect the ability of males to access food in unusual and challenging situations, an therefore be relevant in chick provisioning. Males (4–8 months old) were tested on their ability to obtain a food reward from a clear plastic box with two compartments separated by a sliding clear plastic barrier (26 × 16 cm, and 2 cm high) (Fig. 1c). Birds had to slide the barrier between the compartments to access the reward. Each bird was tested at three difficulty levels of the task (from lowest to highest): the covered compartment was closed 50%, 75%, and 100%. Individuals were tested over 3 days in three blocks of eight consecutive 5-min trials, each block at a different difficulty level. Overall performance (i.e. performance across trials) in this task was measured as the number of trials in which the bird successfully obtained the reward, weighted by the difficulty level. We also included trials in which attempts to obtain the reward were made (i.e. by pecking any component of the clear plastic barrier) as partial successes (to account for effort) by weighting these trials by an additional factor of 0.25 (chosen arbitrarily). Then problem-solving score was calculated according to Eq. (1).

1 $$Problem-solving\;performance= \Sigma (\#succesful\;trials\;*\;difficulty\;level)+0.25\;*\;(\#trials\;with\;attempts\;*\;difficulty\;level)$$

### Spatial memory task

This task was employed to assess individual ability to remember a spatial arrangement of rewards. The ability to remember the location of food sources would give birds an advantage in providing resources for both their mate during incubation, and to the offspring. The device employed was a clear plastic box (10 × 5 cm, and 5 cm high) with 16 equally-sized compartments around three larger central compartments, in which colored dice were fixed as landmarks (Fig. 1c). Habituated individuals (9–18 months old) were then trained for 8 trials of 45 min each. One piece of millet was placed in 4 randomly selected uncovered compartments of the device. Each individual was trained and tested with a unique random selection of rewarded compartments. Each trial was programmed to last 45 min, however if the bird ate all the rewards within 10 min, it was reduced to 20 min. Tests consisted of one trial per day for a total of 5 days; all trials were video recorded. During a test, the cardboard lids covered all the compartments and four pieces of millet were hidden in the same four compartments that were rewarded during training. Performance in this task was the average number of errors made over the five trials. Number of errors is a standard measure of performance in spatial memory tasks of this nature^25,40^. An error consisted of: (1) A visit to an unrewarded compartment, (2) inspection of an unrewarded compartment, or (3) revisiting a compartment already searched.

### Seed discrimination task

The purpose of this task was to test the ability to discriminate between whole and edible seeds and non-edible seed husks. Budgerigars are ground foragers, and efficiently discriminating seeds (i.e. a perceptual cognitive ability) that have been de-husked from whole seeds would in turn increase their foraging efficiency. Therefore, the seed discrimination task is ecologically relevant in this species. We modified the pebble-seed discrimination task^41^. It consisted of a petri dish with seed husks of different colors, sizes and shapes adhered to it and 50 loose whole seeds (same color, size and shape) scattered (Fig. 1c). Birds were tested in this task at 8–18 months of age. A trial consisted of a period no longer than 10 min in which the bird was allowed to consume seed from the device. Once the bird started pecking the seeds or seed husks, it was allowed to eat for 5 min. Testing was suspended if a bird did not peck at the seeds or husks in the first three tests (one test per day), which was the case for 14 individuals. The rest of the birds were tested five times (*N* = 16). Performance in each trial was measured as the number of seeds consumed in 5 min divided by the total number of pecks in each test and then multiplied by 100 (percent discrimination efficiency). Overall performance was the highest discrimination efficiency achieved over all the trials completed (maximum seed discrimination efficiency).

### Detour-reaching task

We assessed the ability to learn how to obtain a food reward when a transparent barrier was placed between the subject and the reward. In order to successfully complete the task, birds had to learn to circumvent the transparent barrier in order to access the food. This task is commonly used to test inhibitory control^42^ (i.e. in this case, the ability to inhibit the impulse to peck at the barrier to obtain the reward). However, we considered that retrieving the reward by circumventing the barrier is an indicator of the bird’s learning ability, which can greatly impact their ability to obtain resources for their mate and offspring. Thus, our measure of performance in this task is an indicator of learning ability instead of inhibitory control. This test^43^ was conducted at 8–18 months, using a clear plastic cylinder with 2 openings (9 cm in length, 6.5 cm diameter) (Fig. 1c). Tests with this clear cylinder were conducted ten times, one test per day for a maximum of 5 min each. The cylinder was rotated 90° after each test to avoid side biases. Detour-reaching performance (i.e. detour-reaching score) was calculated as the percent of successful trials, those in which the bird reached detour to obtain the reward. The higher the percent of trials in which the bird reached the detour in order to obtain the reward, the higher the ability of the bird to learn that the reward cannot be reached by simply pecking at the cylinder. No score was obtained for 11 individuals that did not peck or eat from the device during testing.

Tasks were presented multiple times, which diluted the potential effect of the individual’s internal state during task presentations. All the individuals were given the same opportunities to solve the tasks. For further details on procedures employed in each cognitive task, see the “Supplementary Information”.

### Calculation of the composite cognitive score

For this score, we split the values for each measure of cognitive performance into four ranks (1–4, with four being the highest quartile) using the distribution quartiles as dividing points. If an individual did not participate in a task, it was assigned a value of 0 for that task. A previous study that included the same males tested in this study showed that personality did not determine an individual’s willingness to participate in the cognitive tasks (i.e. being assigned with a value of 0 for a task), except for the seed discrimination task^22^. The same study also showed that task performance is not affected by the age at which subjects were tested. The composite cognitive score for an individual was the sum of the scores obtained for all tasks, with a potential range from 0 to 16. A composite cognitive score of zero indicates lower cognitive ability. Since all the birds had the same exposure to the tasks, multiple times, it is clear that individuals that did not interact or attempt to solve a task made the decision to do so, which is a cognitive process itself. Therefore, it is expected that these individuals will have a limited ability to deal with cognitively challenging situations, which will in turn impact their ability to raise offspring.

### First stage: female choice for a social mate

During this portion of the study, budgerigars were sexually mature. Males (*N* = 30) were between 18 and 24 months old and females were between 14 and 20 months old (*N* = 15). Male pairing success was determined with a free-pairing experiment with five mixed groups housed in separate flight cages, each composed of six males and three randomly selected females. We chose to use a social free-pairing experiment to test our hypothesis rather than a traditional two-choice paradigm because the free pairing experiment allows females to fully evaluate among a group of six courting males based on such potentially important indicators of male cognitive abilities as courtship feeding or close-range courtship displays^18^, while a traditional choice paradigm provides limited access to only two males. Furthermore, this design allowed us to ensure that we were measuring actual female mate choice as demonstrated by the formation of pair bonds, rather than initial female preferences based on limited or no contact with prospective mates.

Each of our five replicate groups were composed of six males (22 months old on average) and three females (18 months old on average). Males were unfamiliar to females and vice versa and both sexes lacked previous mating experience. Previous studies have suggested that female budgerigars show preferences for male UV plumage reflectance^34,35^. To minimize the possible confounding effects of this preference, we assembled male groups with homogeneous plumage characteristics. Three of the group replicates were composed exclusively by males of wildtype plumage morph (yellow head, bright green body, and heavy black striped pattern on the head); one group was constituted exclusively by males with the grey factor plumage mutation (yellow head, olive green body, and heavy black striped pattern on the head); and one group only had males with the opaline mutation (yellow head, bright green body, and light black striped pattern on the head). Females were chosen randomly from a pool of females exhibiting all three plumage morphs.

We also measured other factors that may influence female mate choice for a social mate in budgerigars such as aggressiveness, sociability, and body condition index of the males (see “Supplementary Information”).

### Second and third stages: female choice for extra-pair mates and reproductive investment

The 45 test subjects were released in an indoor aviary (3.4 × 5.2 m) 45 days after the conclusion of the pairing phase. The aviary was equipped with 21 identical wooden nest boxes. Body mass (to the nearest 0.1 g) and tarsus length (to the nearest 0.01 mm) were measured for all individuals at the beginning of breeding and used to calculate the body condition index. Birds were allowed to breed for 6 months and nest monitored daily (see the “Supplementary Information”). We collected approximately 0.15 ml of blood from the brachial vein on an FTA Elute Micro card (MilliporeSigma, Burlington, MA) from nestlings and adults. Each card was labeled with the individual band numbers and stored in an individual envelope at room temperature.

### Molecular parental analysis

We amplified eight microsatellite regions using the polymerase chain reaction (PCR). We amplified two previously developed microsatellite markers (BGMSAT14 and BGMSAT18)^44^ and developed six additional microsatellite markers using the budgerigar genome. Details on PCR reactions, microsatellite design and characteristics (Table S10 in the “Supplementary Information”), genotyping, and paternity assignment are provided in the “Supplementary Information”.

### Statistical analyses

Statistical analyses were conducted and data visualizations created in R version 3.2.1 (R: A Language and Environment for Statistical Computing, R Core Team, R Foundation for Statistical Computing, 2021, Vienna, Austria, https://www.R-project.org). We checked for collinearity among performance in the problem-solving, spatial memory, seed discrimination, and detour-reaching tasks using Spearman correlations.

To test the effects of male cognitive performance, personality, aggressiveness, sociability, and body condition on male pairing success (i.e. first stage of female mate choice), we ran a generalized mixed effects model (GLMM) with binomial distribution using the ‘lme4’ package for R. Male pairing success was entered as the dependent variable and group replicate number was entered as a random factor. Composite cognitive score, aggressiveness, sociability, personality score (see the “Supplementary Information”), and body condition index (BCI) (see the “Supplementary Information”) were entered as fixed factors. Before running the model, we verified that all the factors included in the model were not collinear. The composite cognitive score was not strongly associated with either male BCI (Spearman rank correlation: *N* = 30, *R* = − 0.334, *P* = 0.224) or plumage (Kruskal–Wallis rank sum test: *H* = 1.667, *d.f.* = 2, *P* = 0.435). No association was found between male BCI and plumage morph (Kruskal–Wallis rank sum test: *H* = 0.121, *d.f.* = 2, *P* = 0.941). Male composite cognitive score was negatively correlated with personality score (Spearman rank correlation: *N* = 30, *R* = -0.583, *P* = 7.24 × 10^–4^), with bold and highly exploratory birds obtaining a low cognitive score, and shy and un-exploratory birds obtaining high cognitive scores. We ran all the models with two data sets: (1) all males, excluding the variables aggressiveness and sociability, in order to include six individuals for which values for those variables were missing; and (2) only males with complete data for all factors.

To test female mate choice at the second stage and female reproductive output, we calculated four measures of reproductive success (dependent variables) across multiple nesting attempts during breeding for each male. One measure, total number of nestlings sired, included both within and extrapair genetic offspring. A second measure was the number of extrapair offspring only. The other measure was based only on the nestlings for which a male was the social father, the total number of nestlings fledged. The number of nestlings that actually fledged rather than just the number of nesltings that a male produced is an adequate indicator of a male’s provisioning, which is a direct consequence of cognitive processes underlying resource acquisition.

To test the effects of male cognitive performance and other covariates such as male BCI and plumage morph on the total number of nestlings sired and fledged, and extrapair nestlings, we ran univariate generalized linear models (GLMs) with Poisson distribution. Before running the models, we confirmed that the independent variables were not collinear. For the models in which the number of nestlings sired was the dependent variable, all males were included (i.e. paired and unpaired males), whereas for the models in which the dependent variable was number of extra-pair nestlings, only paired males were included.

Models that included total number of nestlings as the response variable were fitted with: (1) the full data set of 15 males that performed nesting attempts (i.e. paired males), and (2) a subset that excluded two males that nested with their respective mates in the same box, and another two males that were paired and nested with the same female (four males excluded in total, referred as ‘males with shared parental care’). For each pair of males with shared parental care in model set (1), the total number of nestlings that fledged from their nests was split in half between the two males in the full data set.

We included the following variables as measures of female reproductive investment (third stage of choice): (1) Total number of eggs laid, (2) eggs laid in the first nesting attempt, (3) eggs laid in subsequent nesting attempts, and (4) total number of nesting attempts. We fitted GLMs to test the effects of male cognitive performance on the total number of eggs laid and total number of nesting attempts. In order to test differences in the number of eggs laid between the first and subsequent nesting attempts, we fitted GLMs with a Poisson distribution, including an interaction term between nesting attempt (i.e. first and subsequent attempts), and given measure of cognitive performance.

## Supplementary Information

Supplementary Information.

## Data Availability

All data needed to evaluate the conclusions in the paper are present in the paper and/or the “Supplementary Materials”. Additional data, and data required to reproduce the results presented in this study are available in Figshare: https://figshare.com/s/82d56c79c8dd1cc754b7.
