# Supplementary material for: An integrative measure of cognitive performance, but not individual task performance, is linked to male reproductive output in budgerigars
Source: Sci Rep. 2021 Jun 3;11:11775. doi: 10.1038/s41598-021-91213-3 (PMC8175410; doi:10.1038/s41598-021-91213-3)
Supplement: Supplementary file 1 — Supplementary Information. [file 41598_2021_91213_MOESM1_ESM.pdf]

Supplementary Information for:

An integrative measure of cognitive performance, but not individual task performance is linked to male reproductive output in budgerigars

A. Medina-García*, T. F. Wright

* Corresponding author

Contents:

Supplementary Materials and Methods

Supplementary Figure 1. Effect of male cognitive performance on eggs laid in the first and subsequent nesting attempts.

Supplementary Table 1. Summary statistics for measures of performance in cognitive tasks, composite cognitive score, aggression, sociability, personality score, and body condition index in male budgerigars.

Supplementary Table 2. Variation in composite cognitive score and ranks for each task of male budgerigars by group (test of social mate choice).

Supplementary Table 3. Generalized linear mixed models to evaluate the effect of task performance, personality, and body condition on male pairing success.

Supplementary Table 4. Effects of male performance in each task, male plumage morph, and male body condition index (BCI) on number of nestlings sired.

Supplementary Table 5. Effects of male performance in each task, male plumage morph, and male BCI on number of extra-pair nestlings.

Supplementary Table 6. Effects of measures of male cognitive performance on the number of eggs laid, in function of nesting attempt (first attempt vs subsequent attempts).

Supplementary Table 7. Effects of composite cognitive score and male performance in each task, male plumage morph, and male BCI on: A) Number of nesting attempts, and B) total number of eggs laid.

Supplementary Table 8. Effects of male composite cognitive score, male plumage morph, and male body condition index (BCI) the number of nestlings fledged.

Supplementary Table 9. Effects of male performance in each task, male plumage morph, and male BCI on the number of nestlings fledged.

Supplementary Table 10. Characteristics of microsatellite loci amplified from *Melopsittacus undulatus*.

References

Supplementary Materials and Methods

Pre-experimental housing conditions and experimental setup

Males were tested for cognitive ability between 3–18 months of age, before their pairing success was tested as adults. We have previously found that male age does not affect cognitive performance in budgerigars [1]. At the beginning of the test series for cognition, each male was transferred to an individual cage (38 x 27 cm, and 30 cm high) with a parakeet toy, cuttlebone, and water available *ad libitum*. Once in these cages, birds were kept at 90% of their free-feeding weight by only providing limited amount of food for 3 hours in the afternoon to motivate food consumption during tests in the following morning. Tests were conducted between 0800 and 1200 hours, 5–7 days a week, once a day for each individual. The test cage was a plexiglass enclosure (31.5 x 48 cm, and 46 cm high), equipped with a single door (21 x 21 cm) used to introduce test subjects into the cage. This cage was housed in a closed room (3.4 x 1.7 m) with fluorescent lighting and a surveillance camera system (CCTV Security Pros LLC, Cherry Hill, NJ) that allowed the researchers to monitor the activity of the subjects during the tests. Each

individual was transferred from its housing cage to the test cage for testing and then transferred back to the housing cage once the test for that day ended.

Each male was also tested for exploratory behavior and neophobia before being tested in the battery of cognitive tests. Exploratory behavior was assessed as the proportion of regions marked on the test cage floor that the bird visited within 20 min.

We conducted this test of exploratory behavior the first day that birds were exposed to the test cage and again the following day. We tested individual response to 3 unfamiliar objects (problem-solving and seed discrimination device, and a small plastic statue of a red dragon next to a familiar food dish) and one novel food item (commercial papaya yogurt treats for parakeets). Neophobia was measured as the latency in seconds to first peck at the food or the object within a 20 min trial, which took place in the test cage. A Principal Components Analysis was conducted on all measures of exploration and neophobia and the first principal component, which explained 49.26% of the variance and reflected both exploratory and neophobia, was used as a personality score for subsequent analyses. Individuals with low values for this score were shy and little exploratory, whereas birds with high scores were bold and highly exploratory). For more details on personality tests in budgerigars and test correlations, see [1].

All the birds were tested in the problem-solving task first. The order of testing for the other three tasks was not randomized, and it was rather dictated by logistics. However,

we did not find a significant effect of testing order on performance in the spatial memory task (linear model (LM): $t = -0.315$, $b = 1.073$, $P = 0.757$), or the detour-reaching task (linear model (LM): $t = -1.761$, $b = 1.238$, $P = 0.097$), or the seed discrimination task (linear model (LM): $t = -0.914$, $b = 64.064$, $P = 0.376$).

Problem-solving task

Habituation: Before testing, individuals were habituated to the problem-solving device alone first and then with another male. During habituation, birds had open access to the food in the device (the clear plastic barrier was not present). An individual met the habituation criterion once it consumed food from the device in 3 individual habituation tests. Birds were habituated to the device for a maximum of 8 weeks. Only 3 individuals did not meet the habituation criterion and were not tested in the task.

Shaping: Habituation to the device was followed by a shaping phase, which consisted of progressive reduction of the degree of access to the reward by gradually sliding closed the clear plastic barrier. Each bird was shaped for a total of 20 5-min sessions over 2–4 days and then tested at 3 difficulty levels of the task (Figure 1c). Individuals were tested over 3 days in 3 blocks of 8 consecutive 5-minute trials, each block at a different difficulty level. The first trial of the daily session was preceded by a maximum of 3 min of free access to reward placed inside the device. If a bird consumed food before 3 min,

then it was allowed to eat only for 10 s. Each individual was tested at all difficulty levels, from the lowest to the highest difficulty level.

Spatial memory task

Habituation: Males aged 11–17 months were habituated to the device until they consumed food for 2 days. Individuals that did not eat from the device in 5 days were not tested further ($N = 9$).

Detour-reaching task

Habituation and training: Birds were first habituated in the housing cages to an empty opaque cylinder of identical dimensions of the clear cylinder. Then birds were trained to eat from the opaque cylinder until they consumed millet from the inside of the cylinder 3 times (only one bird failed the training).

Housing and monitoring during the test of female choice for a social mate (First stage)

Each group was housed in a separate closed room (3.4 x 1.7 m) in a wrought iron flight cage (79 x 52 cm, and 135 cm high) under a 14:10 light:dark cycle (lights on at 0600, and off at 2000 hours). The room temperature was maintained at 24 ± 2 ºC under natural spectrum lighting. Humidity was maintained on average between 32–90%. All cages were equipped with multiple wooden perches of different thickness, toys, and parakeet seed mix, cuttlebone, and vitamin water were available *ad libitum*. Birds were fed *ad*

libitum with a commercial breeding formula for parakeets with high protein and fat contents. In order to stimulate pair formation, 4 nest boxes were attached to the cage with free access for the birds.

All individuals in each group were video recorded each day for 15 min (males) or 5 min (females) in randomized order, preceded by a 5 min acclimation period with the experimenters in the room. 2 experimenters sat with a video camera approximately 3 m away from the cage. One experimenter filmed the focal individual to ensure that it remained in the field of view during the entire sampling time. The other experimenter took notes of the interactions observed and the leg bands of the interacting birds to ensure that each individual was correctly identified in case that the leg bands were not visible in the video recording. Video-recording sessions were conducted between 0800 and 1000 hours, 2–5 days a week. A pair was considered as stable if: 1) A male and female were seen copulating during 2 consecutive observation periods, or 2) a female engaged in allopreening with or was allofed by the same male during 2 consecutive observation days. The validity of these criteria for pair stability was confirmed since pairs identified using such criteria remained together for the remainder of the study. All groups were allowed to form stable pairs for a maximum of 10 weeks.

Aggressiveness was quantified based on the interactions mediated by agonistic behaviors; whereas sociability was quantified based on interactions mediated by affiliative behaviors. Affiliative (courtship displays, allofeeding, allopreening, copulation solicitation, and copulation) and agonistic behaviors (displacements, attacks, bill thrusts,

etc.) observed during the pairing experiment were classified from the video recordings using the behavioral descriptions in [2,3] and [4]. Only interactions that occurred one week before the first stable pair was detected (pre-pairing) were used to quantify both aggressiveness and sociability, since these interactions were the ones that were likely to play a role in female mate choice. The widely used social network metric, degree centrality [5], was calculated to estimate the levels of both aggressiveness and sociability of each individual. Degree centrality is the product of the number of individuals that a focal bird interacts with, and the average weight (number of interactions) with these individuals, adjusted by the tuning parameter α. We set α to 0.5 and the degree centrality was divided by the total observation time for each individual, which varied between individuals from different groups. We were not able to obtain measures of aggressiveness or sociability for the 6 males in one group replicate because the first stable pair was observed within the first week that the pairing experiment was initiated. Social network metrics were calculated with the R package 'tnet' version 3.0.14 [6]. Male body mass (to the nearest 0.1 g) and tarsus length (to the nearest 0.01 mm) were measured for all individuals at the beginning of their pairing experiment and used to calculate a body condition index (BCI) by dividing body mass by (tarsus length)^3, and then multiplying by 10^4, due to small values, as done in previous studies [7,8].

Breeding and nest monitoring

Birds were kept under a 14:10 light:dark cycle, under standard fluorescent lighting, at a room temperature of 24 ± 2 ºC. Their diet consisted of parakeet seed mix supplemented with parakeet breeding formula with high fat and protein content. The supplemented seed

mix was provided in six different feeders, in close proximity, all in the same location of the aviary. These feeders were filled with seed mix, and seed husks were removed to prevent the blockage of the feeding trays at least every other day. Some feeders were filled less frequently than others because birds favored the location of some feeders over others. Additional small open seed containers were also offered to the birds in periods of high food demand (i.e. when pairs had nestlings or fledglings). Birds were also introduced to foraging toys throughout the breeding phase of the study, placed in different locations of the aviary. The foraging toys were filled with millet only, a highly preferred food item by this species. Birds were also provided with parakeet toys, cuttlebone, and water available *ad libitum*.

The contents of each nest box were checked and recorded three times a week immediately after the conclusion of the morning observation session. Each egg found in the nest boxes was marked with a number with a soft lead pencil in numerical sequence. Eggs were candled in order to estimate the laying date based on the size of the air sac.

Whenever an egg hatched, we estimated the hatching date based on the last time that the nest box was monitored and the size of the nestling. A non-toxic permanent marker was used to mark each nestling on the right leg with a unique color that indicated its hatching order. This color-coding was consistent across nest boxes. Nestlings were banded with the same system employed for adults approximately at 30 days of age (before fledging).

We monitored from beginning to end a total of 29 nests (i.e. nesting attempts) and collected DNA from 83 nestlings throughout the 6-month breeding period.

PCR reactions, microsatellite design, and genotyping

Parental and offspring genomic DNA was extracted from the cards following the manufacturer's protocol with minor modifications (Whatman, Maidstone, UK). Genomic DNA from dead nestlings was extracted from tissue samples with DNAeasy extraction kits following manufacturer's protocol (Germantown, MD).

PCR reactions contained a total of 15 µl with final concentrations of 1X PCR buffer, 8 mM of deoxynucleoside triphosphates, 2.0–3.0 mM of MgCl2, 10 µM of each primer, 2 µl at a 1:2 to 1:5 concentrations of DNA template, and 0.54 U of AmpliTaq Gold polymerase (Applied Biosystem, Foster City, CA), and ultrapure water to volume. Magnesium concentrations were optimized for each locus. PCR was performed on a PTC-200 Thermocycler (MJ Research, Waltham, MA) with a profile of 95°C for 8 min followed by 34 cycles of 95°C for 25 s, 52°C for 30 s, and 72°C for 45 s, followed by 5 min at 72°C. PCR reactions for each sample were combined and purified using the Qiaquick 96 PCR Purification Kit Vacuum protocol (Qiagen, Germantown, MD). Samples were processed for fragment analysis on an ABI 3130XL using LIZ-600 size

standard at the University of Texas El Paso's Genomic Analysis Core Facility. Genotypes were scored visually using Geneious 9.0.5 (http://www.geneious.com) [9].

We searched for microsatellite regions by scanning the *M. undulatus* genome downloaded from https://www.ncbi.nlm.nih.gov/genome/?term=melopsittacus. We used the software Geneious version 9.0.5 (http://www.geneious.com) [9] to find these microsatellite regions in the budgerigar genome. Primers for 27 microsatellite regions were designed with the software Primer3 0.4.0 [10, 11] with PCR product lengths between 150 and 400 base pairs. Only 6 from the 27 regions tested, amplified satisfactorily and also showed actual microsatellite markers after confirmation via sequencing.

We tested for Hardy-Weinberg equilibrium using Genepop version 4.2 [12]. Presence of null alleles was tested with Cervus 3.0.7 [13]. From the 8 microsatellite markers amplified, 7 were in the Hardy-Weinberg equilibrium and their maximum frequency of null alleles was 6% (Table S8). The marker BGMSAT14 was excluded from paternity analyses because it was not in the Hardy-Weinberg equilibrium, suggesting null alleles (Table S10). The mean expected heterozygosity across the remaining 7 loci was 0.61 and the observed heterozygosity was 0.

We used Cervus 3.0.7 to assign paternity to the most likely father under both relaxed (80%) and strict (95%) confidence levels by calculating likelihood ratio scores (LODs). Critical values for these scores were estimated with simulations using the following custom parameters: 30 candidate fathers and 0.99 proportion of loci typed. We simulated

10,000 offspring genotypes using default parameters in Cervus. We ran the 'paternity analysis' option since the known mothers were confirmed by our observations, except for 12 offspring for which the mother was unknown because two females laid eggs and raised these offspring in the same nest box. Paternity analyses were carried out in two steps: in the first step, we included as candidate fathers all 30 males. We excluded 8 offspring in this step because they were typed at fewer than 4 loci. We assigned a father to 61 offspring in the first step. The combined non-exclusion probability for this first round was 0.063 for the first parent and 0.009 for the second parent when the first was known. The nestlings that: 1) were not assigned a father with either 80% or 95% of confidence, and 2) had a known mother, were then selected for the second step ($N = 14$). In this step, the pool of candidate fathers was then reduced to the female's mate and any other males that were seen copulating with the female during our daily observations. Copulations were a common event during these observations and females allowed copulation only with few males. The total number of nestlings that met the 80% or 95% confidence criterion after both the first and second steps was 72. This final subset of nestlings was then employed in further statistical analyses to assess the effects of male cognition on number of nestlings sired and extrapair nestlings.

Supplementary Figures

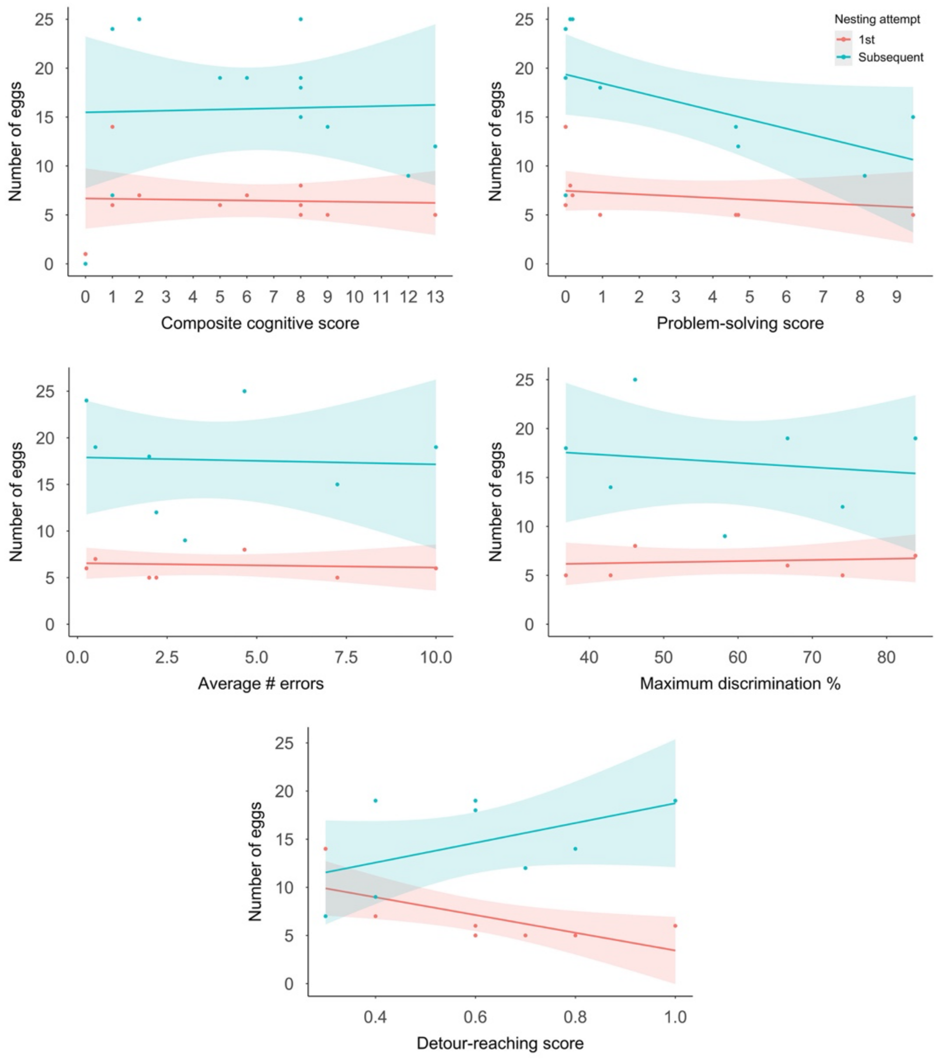

Supplementary Figure 1. Effect of male cognitive performance on eggs laid in the first and subsequent nesting attempts. Measures of male cognitive performance shown are the composite cognitive score, problem-solving score, average number of errors in the spatial memory task, % maximum discrimination in the seed discrimination task, and detour-reaching score. The number of eggs that the female laid in the first nesting attempt

correspond to the red line, and the number of eggs laid in subsequent nesting attempts correspond to the blue line.

Supplementary Tables

Supplementary Table 1. Summary statistics for measures of performance in cognitive tasks, composite cognitive score, aggression, sociability, personality score, and body condition index in male budgerigars.

Measure	N	Mean ± SE	Range
Problem-solving score	27	2.498 ± 0.763	0 – 14.5
Average # errors (spatial memory task)	19	2.214 ± 0.602	0 – 10
Maximum discrimination % (seed disc. task)	16	51.403 ± 4.215	16.667 – 83.871
Detour-reaching score	18	0.661 ± 0.050	0.3 – 1
Composite cognitive score	30	5.9 ± 0.733	0 – 13
Aggression	24	0.161 ± 0.024	0.058 – 0.517
Sociability	24	0.156 ± 0.017	0.019 – 0.451
Personality score	30	0 ± 0.314	-2.637 – 2.546
Body condition index	30	148.906 ± 4.132	109.660 – 197.516

Supplementary Table 2. Variation in composite cognitive score and ranks for each task of male budgerigars by group (test of social mate choice). * Indicates the male that first formed a stable pair in the group.

Male ID	Group #	Composite cognitive score	Problem-solv. rank	Spatial mem. rank	Seed disc. rank	Detour-reach. rank
152	1	0	0	0	0	0
160*		2	2	0	0	0
141		5	2	1	1	1
130		6	0	4	1	1
121		8	0	0	4	4
158		8	3	1	1	3
132	2	2	2	0	0	0
150		5	0	4	1	0
143		8	3	2	2	1
146		9	4	2	3	0
161		9	0	3	3	3
128*		12	4	3	1	4
144*	3	0	0	0	0	0
140		1	0	0	0	1
133		2	0	0	0	2
118		7	3	0	2	2
120		10	4	2	3	1
151		10	3	3	1	3
155	4	1	0	0	1	0
126		3	3	0	0	0
129		4	0	4	0	0
124*		8	2	2	0	4
142		7	0	1	2	4
122		11	4	0	4	3
131	5	0	0	0	0	0
134		8	4	0	0	4
135		4	3	0	0	1
138		1	0	0	1	0
139		12	4	3	4	1
149*		13	4	4	2	3

Supplementary Table 3. Generalized linear mixed models to evaluate the effect of task performance, personality, and body condition on male pairing success. Results from the model with the composite cognitive score as the independent variable are shown in the main text. BCI: body condition index.

All males included:					
Task	Variable	Estimate	SE	*Z* value	*P*-value
Problem solving	Intercept	0.765	0.509	1.505	0.132
	Problem-solving score	-0.108	0.121	-0.894	0.371
	Personality score	0.604	0.511	1.183	0.237
	BCI	-0.056	0.452	-0.123	0.902
Spatial memory	Intercept	0.832	0.580	1.434	0.151
	Average # errors	-0.695	0.603	-1.151	0.250
	Personality score	1.066	0.701	1.520	0.129
	BCI	-0.521	0.557	-0.934	0.350
Seed discrimination	Intercept	0.210	0.636	0.331	0.741
	Max. seed discrimination %	-0.840	0.669	-1.254	0.210
	Personality score	0.431	0.664	0.649	0.516
	BCI	-0.118	0.551	-0.214	0.831
Detour reaching	Intercept	-3.090	2.168	-1.425	0.154
	Detour-reaching score	5.766	3.531	1.633	0.102
	Personality score	0.498	0.836	0.595	0.552
	BCI	0.770	0.794	0.970	0.332
Six males excluded:					
Task	Variable	Estimate	SE	*Z* value	*P*-value
Problem solving	Intercept	1.278	0.715	1.788	0.074
	Problem-solving score	-0.245	0.180	-1.361	0.173
	Personality score	1.073	0.742	1.445	0.149
	Aggressiveness	0.081	0.557	0.145	0.884
	Sociability	-0.601	0.592	-1.016	0.310
	BCI	-0.607	0.666	-0.911	0.362
Spatial memory	Intercept	1.076	0.776	1.387	0.166
	Average # errors	-0.900	0.956	-0.941	0.347
	Personality score	2.113	1.367	1.545	0.122

	Aggressiveness	-0.211	0.587	-0.360	0.719
	Sociability	-0.488	0.548	-0.891	0.373
	BCI	-1.724	1.059	-1.628	0.104
Seed discrimination	Intercept	0.171	0.797	0.215	0.830
	Max. seed discrimination %	-0.985	0.930	-1.059	0.289
	Personality score	1.188	0.964	1.232	0.218
	Aggressiveness	-0.120	0.630	-0.191	0.848
	Sociability	-0.196	0.831	-0.236	0.813
	BCI	-1.001	0.817	-1.225	0.221
Detour reaching	Intercept	3.313	4.428	0.748	0.454
	Detour-reaching score	-2.805	6.220	-0.451	0.652
	Personality score	1.674	1.240	1.350	0.177
	Aggressiveness	-0.976	0.821	-1.188	0.235
	Sociability	-1.024	0.810	-1.264	0.206
	BCI	0.041	1.064	0.039	0.969

Supplementary Table 4. Effects of male performance in each task, male plumage morph, and male body condition index (BCI) on number of nestlings sired. Significant results are indicated in bold. Results from the model with the composite cognitive score as the independent variable are shown in the main text.

Task	*N*	Variable	Estimate (mean ± SE)	*Z* value	*P*-value
Problem solving	27	Intercept	0.740 ± 1.023	0.724	0.469
		**Problem-solving score**	**0.101 ± 0.030**	**3.326**	**0.001**
		Male plumage morph (OP)	-0.031 ± 0.424	-0.073	0.942
		Male plumage morph (WT)	-0.348 ± 0.395	-0.88	0.379
		BCI	2.630×10^{-4} ± 0.007	0.040	0.968
Spatial memory	19	Intercept	1.585 ± 0.955	1.66	0.097
		Average # errors	0.044 ± 0.057	0.771	0.441
		Male plumage morph (OP)	0.948 ± 0.498	1.902	0.057
		Male plumage morph (WT)	0.569 ± 0.428	1.329	0.184
		BCI	-0.008 ± 0.006	-1.307	0.191
Seed discrimination	16	Intercept	2.197 ± 1.217	1.806	0.071
		Max. seed discrimination %	0.001 ± 0.010	0.11	0.912
		Male plumage morph (OP)	0.184 ± 0.450	0.409	0.683
		Male plumage morph (WT)	0.252 ± 0.438	0.575	0.565
		BCI	-0.010 ± 0.006	-1.488	0.137
Detour reaching	18	**Intercept**	**5.926 ± 1.524**	**3.888**	**1.010×10^{-4}**
		Detour-reaching score	-1.421 ± 0.890	-1.597	0.110
		Male plumage morph (OP)	0.619 ± 0.464	1.335	0.182
		Male plumage morph (WT)	0.179 ± 0.465	0.385	0.700
		**BCI**	**-0.032 ± 0.009**	**-3.698**	**2.180×10^{-4}**

Supplementary Table 5. Effects of male performance in each task, male plumage morph, and male BCI on number of extra-pair nestlings. Only paired males were included in these models. Significant results are indicated in bold. Results from the model with the composite cognitive score as the independent variable are shown in the main text.

Task	*N*	Variable	Estimate (mean ± SE)	*Z* value	*P*-value
Problem solving	12	Intercept	-0.389 ± 1.286	-0.302	0.762
		Problem-solving score	0.061 ± 0.049	1.266	0.206
		Male plumage morph (OP)	0.131 ± 0.693	0.189	0.850
		Male plumage morph (WT)	0.630 ± 0.600	1.05	0.294
		BCI	0.005 ± 0.008	0.557	0.577
Spatial memory	11	Intercept	0.440 ± 1.113	0.395	0.693
		Average # errors	0.034 ± 0.075	0.448	0.654
		Male plumage morph (OP)	1.237 ± 0.849	1.458	0.145
		**Male plumage morph (WT)**	**1.319 ± 0.636**	**2.072**	**0.038**
		BCI	-0.004 ± 0.007	-0.597	0.551
Seed discrimination	8	Intercept	0.201 ± 1.548	0.130	0.897
		Max. seed discrimination %	0.005 ± 0.015	0.326	0.745
		Male plumage morph (OP)	0.316 ± 0.714	0.442	0.658
		Male plumage morph (WT)	1.007 ± 0.654	1.539	0.124
		BCI	-0.001 ± 0.006	-0.119	0.906
Detour reaching	9	Intercept	-2.221 ± 4.200	-0.529	0.597
		Detour-reaching score	0.675 ± 1.615	0.418	0.676
		Male plumage morph (OP)	0.698 ± 0.714	0.977	0.328
		Male plumage morph (WT)	1.624 ± 0.893	1.818	0.069
		BCI	0.013 ± 0.023	0.542	0.588

Supplementary Table 6. Effects of measures of male cognitive performance on the number of eggs laid, in function of nesting attempt (first attempt vs subsequent attempts). Only paired males were included in these models. Significant results are indicated in bold.

Measure of cognitive performance	*N*	Variable(s)	Estimate (mean ± SE)	*Z* value	*P*-value
Composite cognitive score	13	**Intercept**	**1.899 ± 0.198**	**9.571**	**< 2 x 10^{-16}**
		Composite cognitive score	-0.005 ± 0.027	-0.199	0.842
		**Subsequent attempts**	**0.841 ± 0.237**	**3.552**	**3.383 x 10^{-4}**
		Composite cognitive score * Nesting attempt (1st and subsequent)	0.009± 0.032	0.284	0.777
Problem solving	11	**Intercept**	**2.011 ± 0.139**	**14.485**	**< 2 x 10^{-16}**
		Problem-solving score	-0.027 ± 0.035	-0.769	0.442
		**Subsequent attempts**	**0.957 ± 0.164**	**5.851**	**4.90 x 10^{-9}**
		Problem-solving score * Nesting attempt (1st and subsequent)	-0.034 ± 0.043	-0.787	0.431
Spatial memory	8	**Intercept**	**1.880 ± 0.215**	**8.763**	**< 2 x 10^{-16}**
		Average # errors	-0.007 ± 0.044	-0.168	0.866
		**Subsequent attempts**	**1.005 ± 0.251**	**4.013**	**6.00 x 10^{-5}**
		Average # errors * Nesting attempt (1st and subsequent)	0.003 ± 0.052	0.061	0.951
Seed discrimination	7	**Intercept**	**1.751 ± 0.563**	**3.109**	**0.002**
		Max. seed discrimination %	0.002 ± 0.009	0.203	0.839
		Subsequent attempts	1.216 ± 0.661	1.839	0.066
		Max. seed discrimination % * Nesting attempt (1st and subsequent)	-0.005 ± 0.011	-0.424	0.671
Detour reaching	8	**Intercept**	**2.750 ± 0.377**	**7.295**	**2.99 x 10^{-13}**
		Detour-reaching score	-1.383 ± 0.655	-2.111	0.035
		Subsequent attempts	-0.490 ± 0.469	-1.045	0.296
		**Detour-reaching score % * Nesting attempt (1st and subsequent)**	**2.069 ± 0.776**	**2.667**	**0.008**

Supplementary Table 7. Effects of composite cognitive score and male performance in each task, male plumage morph, and male BCI on: A) Number of nesting attempts, and B) total number of eggs laid. Only paired males were included in these models. Significant results are indicated in bold.

**A) Dependent variable: Number of nesting attempts**					
Measure of cognitive performance	*N*	Variable	Estimate (mean ± SE)	*Z* value	*P*-value
Composite cognitive score	15	Intercept	1.832 ± 0.958	1.913	0.056
		Composite cognitive score	0.014 ± 0.035	0.410	0.682
		Male plumage morph (OP)	-0.044 ± 0.389	-0.114	0.910
		Male plumage morph (WT)	-0.173 ± 0.307	-0.565	0.572
		BCI	-0.004 ± 0.006	-0.585	0.559
Problem solving	12	Intercept	2.672 ± 1.188	2.249	0.025*
		Problem-solving score	-0.030 ± 0.041	-0.724	0.469
		Male plumage morph (OP)	0.017 ± 0.419	0.040	0.968
		Male plumage morph (WT)	-0.181 ± 0.393	-0.462	0.644
		BCI	-0.008 ± 0.008	-1.04	0.298
Spatial memory	9	**Intercept**	**1.998 ± 0.967**	**2.066**	**0.039**
		Average # errors	0.013 ± 0.051	0.251	0.802
		Male plumage morph (OP)	-0.834 ± 0.771	-1.082	0.279
		Male plumage morph (WT)	-0.320 ± 0.358	-0.894	0.371
		BCI	-0.003 ± 0.007	-0.472	0.637
Seed discrimination	8	Intercept	2.289 ± 1.201	1.905	0.057
		Max. seed discrimination %	$-9.179 \times 10^{-5} \pm 0.011$	-0.008	0.993
		Male plumage morph (OP)	-0.113 ± 0.443	-0.255	0.799
		Male plumage morph (WT)	-0.241 ± 0.458	-0.527	0.598
		BCI	$-5.47 \times 10^{-3} \pm 6.59 \times 10^{-3}$	-0.83	0.407
Detour reaching	9	Intercept	3.685 ± 2.745	1.343	0.179
		Detour-reaching score	0.379 ± 0.966	0.392	0.695
		Male plumage morph (OP)	-0.166 ± 0.394	-0.420	0.674
		Male plumage morph (WT)	-0.801 ± 0.626	-1.280	0.201
		BCI	-0.017 ± 0.016	-1.017	0.309
**B) Dependent variable: Total number of eggs laid**					
Measure of cognitive performance	*N*	Variable	Estimate (mean ± SE)	*Z* value	*P*-value

Composite cognitive score	13	**Intercept**	**2.248 ± 0.381**	**5.908**	**3.46 x 10^{-9}**
		Composite cognitive score	-0.001 ± 0.016	0.040	0.968
		Male plumage morph (OP)	-0.051 ± 0.172	-0.293	0.770
		Male plumage morph (WT)	0.066 ± 0.138	0.481	0.630
		**BCI**	**-0.006 ± 0.003**	**2.212**	**0.027**
Problem solving	11	**Intercept**	**2.732 ± 0.404**	**6.758**	**1.4 x 10^{-11}**
		Problem-solving score	-0.039 ± 0.022	-1.747	0.081
		Male plumage morph (OP)	-0.120 ± 0.187	-0.641	0.522
		Male plumage morph (WT)	-0.058 ± 0.153	-0.381	0.704
		BCI	0.004 ± 0.003	1.494	0.135
Spatial memory	8	**Intercept**	**2.370 ± 0.412**	**5.756**	**8.6 x 10^{-9}**
		Average # errors	-0.010 ± 0.023	-0.419	0.676
		Male plumage morph (OP)	-0.452 ± 0.270	-1.676	0.094
		Male plumage morph (WT)	-0.075 ± 0.161	-0.466	0.641
		**BCI**	**0.006 ± 0.003**	**2.246**	**0.025**
Seed discrimination	7	**Intercept**	**2.263 ± 0.687**	**3.296**	**0.001**
		Max. seed discrimination %	0.001 ± 0.005	0.236	0.813
		Male plumage morph (OP)	-0.174 ± 0.194	-0.899	0.369
		Male plumage morph (WT)	-0.139 ± 0.225	-0.617	0.537
		BCI	0.006 ± 0.003	1.771	0.077
Detour reaching	8	**Intercept**	**4.515 ± 1.540**	**2.931**	**0.003**
		Detour-reaching score	-0.310 ± 0.449	-0.691	0.490
		Male plumage morph (OP)	-0.176 ± 0.173	-1.014	0.311
		Male plumage morph (WT)	-0.394 ± 0.251	-1.567	0.117
		BCI	-0.008 ± 0.010	-0.82	0.412

Supplementary Table 8. Effects of male composite cognitive score, male plumage morph, and male body condition index (BCI) on the number of nestlings fledged. Significant results are indicated in bold.

Data Set	Explanatory variables	Estimate (mean ± SE)	*Z* value	*P*-value
All paired males (N = 15)	Intercept	-0.384 ± 0.766	-0.502	0.616
	**Composite cognitive score**	**0.106 ± 0.037**	**2.906**	**0.004**
	Male plumage morph (OP)	-0.536 ± 0.425	-1.263	0.207
	Male plumage morph (WT)	-0.020 ± 0.310	-0.065	0.948
	BCI	0.008 ± 0.005	1.670	0.095
Males with shared parental care excluded (N = 11)	Intercept	-0.169 ± 0.916	0.185	0.854
	**Composite cognitive score**	**0.125 ± 0.043**	**2.927**	**0.003**
	Male plumage morph (OP)	-0.755 ± 0.490	-1.542	0.123
	Male plumage morph (WT)	-0.272 ± 0.356	-0.762	0.446
	BCI	0.005 ± 0.006	0.864	0.388

Supplementary Table 9. Effects of male performance in each task, male plumage morph, and male BCI on the number of nestlings fledged. Only paired males were included in these models. Significant results are indicated in bold.

Measure of cognitive performance	*N*	Variable	Estimate (mean ± SE)	*Z* value	*P*-value
Problem solving	12	Intercept	0.620 ± 0.921	0.673	0.501
		Problem-solving score	0.025 ± 0.041	0.619	0.536
		Male plumage morph (OP)	-0.649 ± 0.444	-1.463	0.144
		Male plumage morph (WT)	-0.331 ± 0.356	-0.928	0.353
		BCI	0.008 ± 0.006	1.257	0.209
Spatial memory	9	Intercept	1.083 ± 0. 750	1.444	0.149
		**Average # errors**	**-0.155 ± 0.063**	**-2.466**	**0.014**
		Male plumage morph (OP)	-0.279 ± 0.517	-0.54	0.589
		Male plumage morph (WT)	-0.108 ± 0.310	-0.349	0.727
		BCI	-0.008 ± 0.005	1.516	0.129
Seed discrimination	8	Intercept	0.975 ± 1.003	0.973	0.331
		Max. seed discrimination %	0.007 ± 0.009	0.784	0.433
		**Male plumage morph (OP)**	**-0.834 ± 0.410**	**-2.033**	**0.042**
		Male plumage morph (WT)	-0.310 ± 0.359	-0.864	0.387
		BCI	0.005 ± 0.005	0.939	0.348
Detour reaching	9	Intercept	1.559 ± 3.001	0.52	0.603
		Detour-reaching score	-0.583 ± 1.015	-0.574	0.566
		Male plumage morph (OP)	-0.578 ± 0.402	-1.438	0.15
		Male plumage morph (WT)	-0.393 ± 0.558	-0.704	0.481
		BCI	0.004 ± 0.018	0.22	0.826

Supplementary Table 10. Characteristics of microsatellite loci amplified from *Melopsittacus undulatus*. The parameters shown were obtained with Cervus, except for P(HWE) which was calculated with Genepop including only parents. H_O: observed heterozygosity, H_E: expected heterozygosity, NE-1P: average non-exclusion probability for one candidate parent, NE-2P: average non-exclusion probability for one candidate parent given the genotype of a known parent of the opposite sex, P(HWE): probability of Hardy-Weinberg equilibrium, F(Null): estimated null allele frequency. * Indicates probabilities of locus being in HWE equilibrium.

Locus name	H_O	H_E	NE-1P	NE-2P	P(HWE)	F(Null)
BGMSAT14	0.355	0.484	0.884	0.791	0.029*	0.157
BGMSAT18	0.653	0.694	0.736	0.571	0.343	0.022
MUMSAT10	0.667	0.675	0.738	0.567	0.880	-0.006
MUMSAT20	0.770	0.826	0.524	0.351	0.447	0.035
MUMSAT25	0.217	0.213	0.978	0.894	1.000	-0.022
MUMSAT34	0.771	0.861	0.442	0.282	0.856	0.055
MUMSAT37	0.786	0.827	0.518	0.346	0.837	0.026
MUMSAT59	0.174	0.191	0.982	0.91	1.000	0.062

References

1. Medina-García, A., Jawor, J. M. & Wright, T. F. Cognition, personality, and stress in budgerigars, *Melopsittacus undulatus*. *Behav. Ecol.* **28,** 1504–1516 (2017).
2. Brockway, B. F. Ethological studies of the budgerigar (*Melopsittacus undulatus*): reproductive behavior. *Behaviour* **23,** 294–324 (1964).

3. Brockway, B. F. Ethological Studies of the Budgerigar (Melopsittacus Undulatus): Non-Reproductive Behavior. *Behaviour* **22,** 193–222 (1964).

4. Abbassi, P. & Burley, N. T. Nice guys finish last: same-sex sexual behavior and pairing success in male budgerigars. *Behav. Ecol.* **23,** 775–782 (2012).

5. Opsahl, T., Agneessens, F. & Skvoretz, J. Node centrality in weighted networks: Generalizing degree and shortest paths. *Soc. Networks.* **32,** 245–251 (2010).

6. Opsahl, T. Structure and Evolution of Weighted Networks. (Ph.D. Thesis, University of London, 2009).

7. Griggio, M., Zanollo, V. & Hoi, H. UV plumage color is an honest signal of quality in male budgerigars. *Ecol. Res.* **25,** 77–82 (2010).

8. Bókony, V., Liker, A., Lendvai, Á. Z. & Kulcsár, A. Risk-taking and survival in the House Sparrow *Passer domesticus*: are plumage ornaments costly? *Ibis* **150,** 139–151 (2008).

9. Kearse, M. *et al.* Geneious Basic: an integrated and extendable desktop software platform for the organization and analysis of sequence data. *Bioinformatics.* **28,** 1647–1649 (2012).

10. Untergasser, A. *et al.* Primer3—new capabilities and interfaces. *Nucleic Acids Res.* **40,** e115–e115 (2012).

11. Koressaar, T. & Remm, M. Enhancements and modifications of primer design program Primer3. *Bioinformatics.* **23,** 1289–1291 (2007).

12. Rousset, F. genepop'007: a complete re-implementation of the genepop software for Windows and Linux. *Mol. Ecol. Resour.* **8,** 103–106 (2008).

13. Kalinowski, S. T., Taper, M. L. & Marshall, T. C. Revising how the computer program cervus accommodates genotyping error increases success in paternity assignment. *Mol. Ecol.* **16,** 1099–1106 (2007).
